# The role of dietary antioxidants in type 2 diabetes and neurodegenerative disorders: An assessment of the benefit profile

**DOI:** 10.1016/j.heliyon.2022.e12698

**Published:** 2022-12-30

**Authors:** Munazza Tamkeen Fatima, Ajaz Ahmed Bhat, Sabah Nisar, Khalid Adnan Fakhro, Ammira Sarah Al-Shabeeb Akil

**Affiliations:** a Department of Human Genetics-Precision Medicine in Diabetes Prevention Program, Sidra Medicine, P.O. Box 26999, Doha, Qatar; b College of Health and Life Sciences, Hamad Bin Khalifa University, Doha, P.O. Box 34110, Doha, Qatar; c Department of Genetic Medicine, Weill Cornell Medical College, Doha, P.O. Box 24144, Doha, Qatar; d Department of Human Genetics, Laboratory of Genomic Medicine-Precision Medicine Program, Sidra Medicine, P.O. Box 26999, Doha, Qatar

**Keywords:** Antioxidants, Type 2 diabetes, Neurodegenerative diseases, Reactive oxygen species, Diet, Therapeutics

## Abstract

Healthy diet is vital to cellular health. The human body succumbs to numerous diseases which afflict severe economic and psychological burdens on the patient and family. Oxidative stress is a possible crucial regulator of various pathologies, including type 2 diabetes and neurodegenerative diseases. It generates reactive oxygen species (ROS) that trigger the dysregulation of essential cellular functions, ultimately affecting cellular health and homeostasis. However, lower levels of ROS can be advantageous and are implicated in a variety of signaling pathways. Due to this dichotomy, the terms oxidative “eustress,” which refers to a good oxidative event, and “distress,” which can be hazardous, have developed. ROS affects multiple signaling pathways, leading to compromised insulin secretion, insulin resistance, and β-cell dysfunction in diabetes. ROS is also associated with increased mitochondrial dysfunction and neuroinflammation, aggravating neurodegenerative conditions in the body, particularly with age. Treatment includes drugs/therapies often associated with dependence, side effects including non-selectivity, and possible toxicity, particularly in the long run. It is imperative to explore alternative medicines as an adjunct therapy, utilizing natural remedies/resources to avoid all the possible harms. Antioxidants are vital components of our body that fight disease by reducing oxidative stress or nullifying the excess toxic free radicals produced under various pathological conditions. In this review, we focus on the antioxidant effects of components of dietary foods such as tea, coffee, wine, oils, and honey and the role and mechanism of action of these antioxidants in alleviating type 2 diabetes and neurodegenerative disorders. We aim to provide information about possible alternatives to drug treatments used alone or combined to reduce drug intake and encourage the consumption of natural ingredients at doses adequate to promote health and combat pathologies while reducing unwanted risks and side effects.

## 1 Introduction

**Fig. 1:**
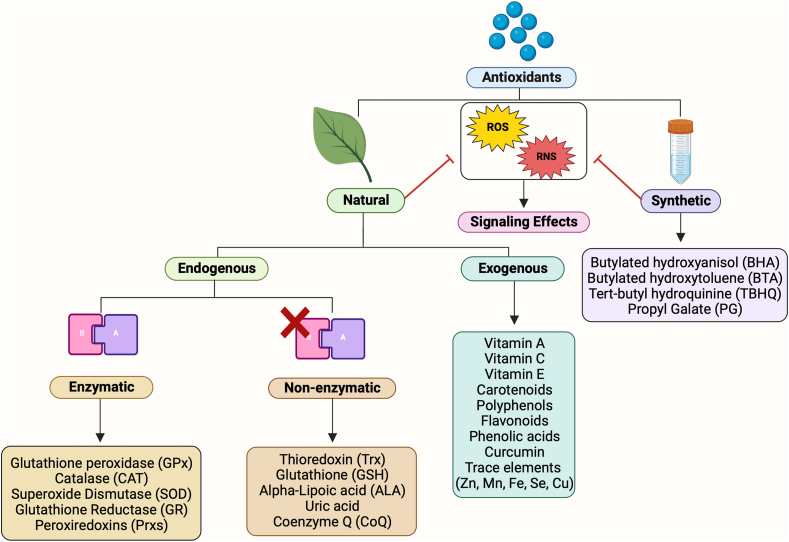
**Classification of antioxidants based on their origin.** This includes natural, endogenous, enzymatic, non-enzymatic, exogenous and synthetic antioxidants that help inhibit the activity of toxic oxidative radicals, including various Reactive Oxygen Species (ROS) and Reactive Nitrogen Species (RNS).

Cellular oxidative stress (OS), which occurs due to a reduction-oxidation (redox) imbalance in the cell, produces reactive free radicals that can damage cells and tissues. Free radicals can have several centers, such as oxygen, nitrogen, sulfur and carbon. These commonly include reactive oxygen species (ROS), e.g., superoxides, singlet oxygen, and hydroxy radicals; reactive nitrogen species (RNS), e.g., nitric oxide and peroxynitrite; reactive sulfur species (RSS), e.g., thyl radicals, disulfide radical; and others (Fig. 1). Metals such as iron and copper play significant roles in the generation of toxic free radicals by involving in many chemical reactions [1]. Endogenous antioxidants help maintain oxidative status by delaying, inhibiting, or preventing oxidation by scavenging free radicals [2,3] and include the enzymes superoxide dismutase (SOD), catalase, and glutathione peroxidase and the non-enzymatic antioxidants such as glutathione, thioredoxin, and peroxiredoxins (Fig. 1) [4,5].

While low to moderate levels of ROS are essential in various physiological processes, their excessive and uncontrolled production causes failure of endogenous defences, leading to oxidative stress (OS) and possibly causing damage to the proteins, lipids, and DNA. Briefly elaborating the concepts of ‘oxidative eustress' and ‘oxidative distress', detailed in Refs. [[6], [7], [8]]-perpetuation of redox homeostasis is a continuous process involving spatiotemporal control of redox signaling, resulting in the generation and removal of oxidants [6]. This dynamic process is referred to as ‘homeodynamics’ and regulates changes at cellular and molecular level. At low physiological levels (nanomolar concentrations), species involved in redox signaling (e.g., H_2_O_2_) are crucial in metabolic regulation and adaptation to any changes in the cellular environment, possibly by being involved in various post-translational modifications, -a state of oxidative eustress. However, a high level of ROS (e.g., above 100 nM in the case of H_2_O_2_) has detrimental effects and causes oxidative distress. We have used oxidative stress/distress in the same context throughout the manuscript.

The role of OS in the pathogenesis and/or complications of numerous diseases is well-established [[9], [10], [11]], including cancer, diabetes, neurological disorders, and cardiovascular issues. It fosters cellular death by activating apoptosis, dysregulating the extra and intracellular matrix proteins and release factors, and disrupts cellular homeostasis by dysregulating multiple cellular signaling pathways. Since scavenging of ROS essentially depends on endogenous and exogenous antioxidant defenses in living organisms, individuals with chronic or degenerative diseases are more susceptible to OS or OS-induced damage due to raised levels of oxidants and/or reduced levels of antioxidants.

Antioxidant supplements have the possible potential to fight conditions linked to OS by scavenging free radicals. Antioxidant drugs are commercially available in the market as multi-vitamin, multi-mineral, antioxidant supplements, and pharmaceutical products containing cysteine, *N*-acetylcysteine, propyl gallate, butylated hydroxytoluene, butylated hydroxyanisole, sodium metabisulfite, lipoic and ascorbic acid [12,13]. Despite their wide use in the pharmaceutical and food industry, they present adverse side effects, including gastric irritation and diarrhea [12]. Reports also suggest that excessive clearance of ROS may be undesirable, possibly damaging specific essential intracellular signaling and metabolic functions and increasing susceptibility to infections [14]. In addition, drugs prescribed against particular diseases are often associated with poor solubility, instability, lack of selectivity, and undesirable side effects. Given the drawbacks of drug-based antioxidative therapies, diet-based approaches present an alternative approach to protecting against OS. A carefully planned diet with an appropriate intake of antioxidant-rich food and physical activity might protect against many pathologies, particularly diabetes, obesity and cardiovascular complications [[15], [16], [17]].

This review will discuss the role, effect, and benefit of dietary antioxidants in type 2 diabetes (T2D) and neurodegenerative diseases. A considerably large population globally carries the burden of these diseases at alarmingly increasing rates. Life style changes, including dietary habits and global revolution in the food industry, as well as aging, may be potential contributors. A common pathological advancement in both these diseases includes increased OS and subsequent impairment/damage. The protective effect may be achieved either by directly neutralizing the ROS/RNS/other free radical species by the transfer of hydrogen atom and sequential proton loss electron transfer, maintaining the cellular redox balance, thereby protecting the cellular milieu from oxidative impairment, or upregulating the expression of cellular defensive genes or regulating various antioxidant signaling pathways. In addition, restraining the source of production of ROS itself, and also upregulating their repair and replacement mechanisms are potential alternatives. Of note, natural dietary supplements can manipulate multiple targets concurrently [[18], [19], [20]] and exert enhanced therapeutic effects by augmenting the effect of specific drugs prescribed for a specific disease [21,22]. Also, otherwise a proper assessment to manage the effect of interactions with medications and supplements is crucial [22,23]. It may be important to point out that despite being associated to many human diseases, and with human studies/clinical trials showing low to moderate efficiency in few cases (detailed subsequently for T2D and neuroprotective effects), effective translation of the modulators of ROS into the clinics is yet highly awaited [24].

### 1.1 Cellular stress and diabetes

**Fig. 2:**
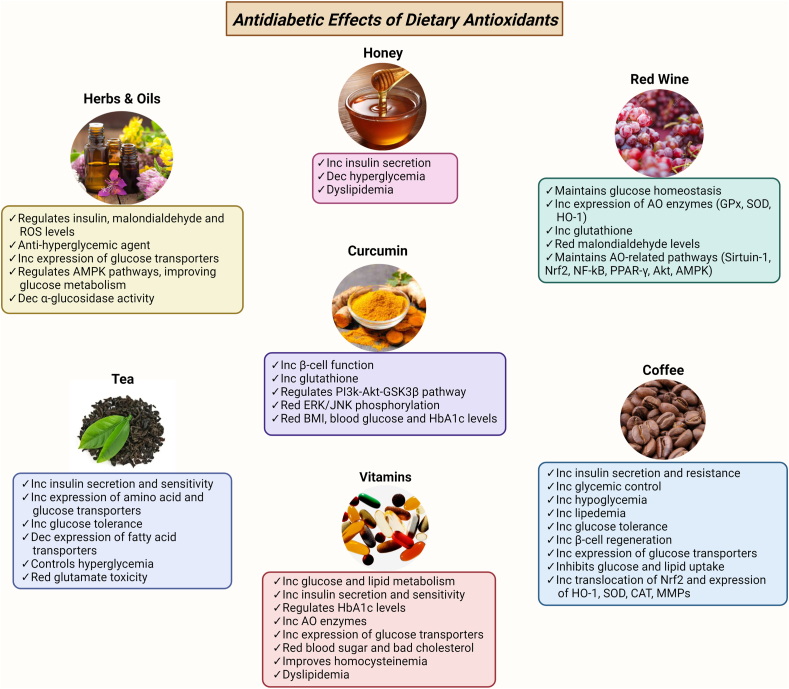
**Precise summary of the effect and action of dietary antioxidants in type 2 diabetes.** These include the effect of Tea, Coffee, Vitamins, Red wine, Honey and common Herbs and Oils, highlighting the precise effect and the pathways that they might target. Inc (increased), Dec (decreased), Red (reduced), AO (antioxidants), Heme oxygenase (HO-1), Superoxide dismutase (SOD), catalase (CAT), matrix metalloproteinases (MMPs), Hemoglobin A1c (HbA1c), nuclear factor erythroid 2-related factor (NRF2), Nuclear Factor kappa B (NF-κB), peroxisome proliferator-activated receptor gamma (PPAR-γ), Protein Kinase B (Akt), AMP-activataed protein kinase (AMPK), extracellular regulated kinase (ERK), c-Jun *N*-terminal linase (JNK), phosphatidylinositol 3-kinase (PI3K), glycogen synthase kinase 3 Beta (GSK3B), ROS (Reactive Oxygen Species). (For interpretation of the references to colour in this figure legend, the reader is referred to the Web version of this article.)

OS contributes to the pathogenesis of T2D and aggravates the pathology and complications by interfering in regulatory pathways involved in insulin resistance and β-cell dysfunction. However, the exact mechanism is not yet understood. T2D and high OS are linked to hyperglycemia, inflammation, and dyslipidemia. Excess ROS acts as a second messenger and regulates the function of important proteins, including Kelch-like ECH-associated protein 1 (Keap1), protein kinase C (PKC), and IκB kinase β by interacting with the cysteine residues (known as redox sensors) and causes their oxidation [10,25]. This oxidization contributes considerably to the T2D pathology, possibly by activating alternative downstream signaling pathways critically involved in insulin resistance and compromised insulin secretion [10]. Current drugs for T2D management include hypoglycemic agents such as metformin, glipizide, glyburide, and tolbutamide administered orally or in combination with insulin injections [26]. These drugs act by increasing glucose transport and insulin sensitivity. Lipid-lowering drugs (lovastatin, pravastatin) and anti-hypertensive drugs (ramipril, chlorthalidone) are also used [26]. Dietary antioxidants that show anti-diabetic effects also improve diabetic status by regulating glucose metabolism, improving insulin secretion and decreasing insulin resistance, improving vascular functions, and regulating the levels of HbA1c and oxidative stress markers (Fig. 2), as detailed in the subsequent sections.

### 1.2 Cellular stress and neurodegenerative diseases

**Fig. 3:**
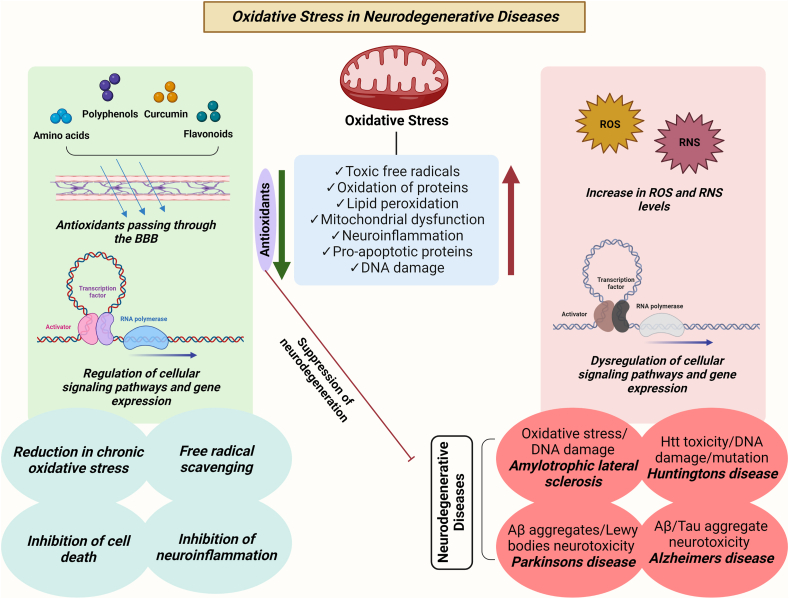
**Overview of the effect of oxidative stress in neurodegenerative diseases and the role of antioxidants.** Upward red arrow (increase), Downward green arrow (decrease), and thin blue arrows indicate the passing of various classes of antioxidant molecules, including polyphenols, flavonoids, amino acids, curcumin, and more across the Blood-brain barrier (BBB), reactive oxygen species (ROS), reactive nitrogen species (RNS). (For interpretation of the references to colour in this figure legend, the reader is referred to the Web version of this article.)

OS is involved in the pathogenesis of several neurodegenerative diseases [27], including Alzheimer's disease (AD), Parkinson's disease (PD), Huntington's disease (HD), multiple sclerosis (MS), and amyotrophic lateral sclerosis (ALS). In these disorders, increased OS can occur due to the exhaustion of antioxidants, mitochondrial dysfunction, glutamatergic excitotoxicity, neuro-inflammation, raised expression of pro-apoptotic proteins, and possible genetic modifications [28]. The elderly population is more susceptible to OS, caused by a deterioration in the efficacy of their endogenous antioxidant system and causes failure of several defense mechanisms to respond to reactive species (ROS, RNS). This affects vital organelles such as mitochondria, causing alterations in the biophysical properties of the membrane, disrupting the electron transport chain activities, decreased fluidity, and subsequently an energy imbalance; and organs such as brain and heart associated with increased oxygen consumption [29]. Considering that origin of most neurodegenerative diseases is sporadic, environmental factors may also regulate OS in neurodegenerative disorders. In most neurological disorders insoluble protein aggregates accumulate in the brain and central nervous system, for example, amyloid ﬁbrils and tau proteins in AD, α-synuclein and Lewy bodies in PD, and Huntingtin-protein aggregates in HD. These aggregates trigger the progressive loss of neuronal structure and function of neurons, resulting in neuronal death [30,31]. Antioxidants suppress neurodegeneration by reducing oxidative damages and toxic free radicals and modulating multiple signaling pathways and gene expression, as evident from the *in vitro* and *in vivo* studies detailed in the subsequent sections (Fig. 3).

### 1.3 Natural antioxidants in common food/beverages and their therapeutic and protective effects

The compounds in dietary food and beverages that reduce OS and modulate cellular defenses are diverse. These include polyphenols, flavonoids, bioflavonoids, and multiple aromatic bio-phenols. Polyphenols are a large group of natural and synthetic small molecules containing one or more aromatic phenolic rings. Natural polyphenols are abundant in fruits, nuts and berries, green leafy vegetables, and other food or beverages, including tea, coffee, olive oil, red wine, and honey [31,32]. Other sources include foods rich in vitamins C, E, and S-carotene and herbs, including thyme, oregano, rosemary, sage, mint, and basil [2] (Table 1). All these can be categorized as nutraceuticals (supplements with health benefits and nutrient value). Polyphenol-based dietary antioxidants ameliorate T2D either by (1) an insulin-dependent approach, *i.e*., by protecting the pancreatic islet β-cell and promoting its proliferation while reducing OS and β-cell apoptosis, which causes activated insulin signaling and secretion (2) an insulin-independent approach, *i.e.,* decreasing the intestinal absorption of glucose, inhibiting the activity of digestive enzymes and regulating glucose discharge from the liver (3) modulating intracellular signaling pathways and (4) modulating gene expression [33] (Fig. 2). Polyphenols are being extensively investigated in cell-based studies, animal models, human studies, and clinical trials as antidiabetic and neuroprotective agents. Polyphenols also inhibit the assembly of various amyloidogenic protein ﬁbrils *in vitro* [34]. They have neuroprotective effects in humans, reducing disease progression by protecting neurons from oxidative damage, Aβ-induced neuronal injury and neurotoxicity, and nitric oxide-induced toxicity while increasing neuronal function and regeneration and modulating neuronal signaling pathways [20,28].

**Table 1:** The therapeutic components of crucial dietary antioxidants including their class, structure, major dietary sources, and proof of evidence based on scientific experiments.

Name/ Category	Chemical structure	Dietary sources	Level of evidence	References
Theanine/ Aminoacid		Green tea, black tea, certain mushrooms	In vitro In vivo Human	[35] [36,37] [38,39]
Catechin/ Polyphenol- Flavanoids (flavan-3-ol)		Green tea, red wine, fruits (apple, various berries, cherries, pear)	In vitro In vivo Human	[40,[41], [42], [43], [44]] [[40], [45], [46],^42^,^47^,[48], [49], [50]] [41,51]
Epicatechin (EC)/ Catechin Derivative		Green tea, red wine, fruits (Apple, various berries)		
Epigallocatechin (EGC)/ Catechin Derivative		Green tea, black tea, fruits (apple, various berries)		
Epicatechin gallate (ECG)/ Catechin Derivative		Green tea, black tea, fruits (apple, various berries)		
Epigallocatechin-3-gallate (EGCG)/ Catechin Derivative		Green Tea, black Tea, fruits (apple, various berries, pear, peaches), nuts		
Caffeine/ Purine (methyl xanthine Alkaloid		Coffee, cocoa, green tea, guarana	In vivo Human	[52] [[53], [54], [55], [56], [57],[58], [59], [60]]
Trigonelline/ Alkaloid (pyridine derivative)		Coffee, barley, vegetables (corn, onions, peas, soybeans), cantaloupe	In vitro In vivo	[61][62,63] [64,65,66,67]
Cafestol/ Diterpenoid		Coffee beans	In vitro In vivo Human	[68,69] [[70], [71], [72]] [72]
Chlorogenic Acid/ Polyphenol (ester of caffeic acid & quinic acid)		Coffee, tea, fruits (apple, berries, pears), honey	In vitro In vivo	[73,74] [75,76,[74], [77], [78]]
Caffeic acid/ Polyphenol (hydroxy cinnamic acid)		Coffee, fruits (apples, berries, olives pear)	–	–
Resveratrol/ Polyphenol- Stilbene		Wine, cocoa, grapes and berries, peanuts	In vitro In vivo Human	[79,80,81] [82,83] [84,85,86]
Quercetin/ Polyphenol- Flavanoids (flavonol)		Red wine, olive oil, fruits, vegetables	In vitro In vivo Human	[[87], [88], [89],^90^,^91^] [91,92,93] [[94], [95], [96],^97^]
Galangin/ Flavanoid- Flavonol		Honey, Galangal root	In vivo Human	[98,99] [100,[101], [102], [103], [104]]
Isorhamnetin/ Flavanoid- flavonol		Honey, herbs, berries		
Hesperetin/ Flavanoid- flavanone		Honey, orange, grapefruit, lemon, tangerines		
Naringenin/ Flavanoid- flavanone		Honey, grapefruit tomatoes, cherries, greek oregano		
Gallic Acid/ Phenolic acid		Honey, fruits including berries, nuts, tea, wine		
Ellagic Acid/ Polyphenol		Honey, fruits including berries, nuts, honey		
Benzoic acid/ Aromatic carboxylic acid		Honey, strawberries, spices, herbs,		
Syringic Acid/ Phenolic compound		Honey, red wine, Grapes, olives, dates		
Cinnamic acid/ Unsaturated carboxylic acid		Honey, cinnamon,		
Myricetin/ Polyphenol Flavanoid		Honey, herbs, berries,		
Coumaric acid/ Hydroxy cinnamic acid		Honey, Fruits, vegetables, tea, coffee, wine		
Chrysin/Flavone		Honey		
Luteolin/ Flavanoid- flavone		Honey, vegetables, fruits, herbs	In vivo	[105,106]
Ferulic acid/ Phenolic phytochemical-Hydroxy cinnamic acid		Honey, whole grains, fruits, herbs	In vivo	[28,107,108]
Kaempferol/ Flavanoid- flavonol		Honey, green leafy vegetables, herbs, ginkgo biloba leaves	In vitro In vivo	[28] [109]
Tulang honey			In vivo	[110]
Apigenin/ Flavone		Honey, herbs, fruits and vegetables, tea, wine	In vitro In vivo	[83] [111]
Vitamin A/Retinol, retinal, retinoic acid (retinoids), carotenes, and xanthins		Meat (liver) and dairy products	In vitro In vivo Human	[112,113,114] [93,97,99,102,106,107] [115,116,117,118,119]
Alpha-carotene/ Carotene (Terpenoid)		Yellow-orange & dark green vegetables		
Beta carotene/Carotene (Terpenoid)		Yellow-orange & dark green vegetables		
Lycopene/ Carotene		Tomatoes, water melon, grapefruits		
Vitamin B1 (Thiamine)/ Essential micro nutrient, water-soluble		Whole grains, legumes, meat, and fish	In vivo Human	[120,121] [122]
Vitamin B2 (Riboflavin)/ Essential micro nutrient, water-soluble		Dairy products, egg, meat, fish	In vitro In vivo Human	[123,124] [123,125,124] [126]
Vitamin B3 (Niacin/Niacinamide)/ Essential nutrient, water soluble		Cereals, milk, egg, meat, fish, green vegetables	In vitro In vivo Human	[108][123,127] [127] [[127], [128], [129], [130], [131], [132]]
Vitamin B6 (Pyridoxine)/Essential nutrient, water-soluble vitamin		Meat (liver), fish, poultry, dark leafy vegetables, fruits (banana, papaya)	In vitro In vivo Human	[133] [134,133] [[135], [136], [137]]
Vitamin B9 (Folic acid)/Essential nutrient, water-soluble vitamin		Dark green leafy vegetables (spinach, asparagus, broccoli, brussels sprout), beans	In vitro In vivo Human	[138,139] [138] [[140], [141], [142]]
Vitamin B12 (Cobalamin) Essential nutrient, water-soluble vitamin		Dairy, meat, fish		
Vitamin C (Ascorbic acid)/ nutrient, water-soluble vitamin		Citrus fruits, cruciferous vegetables (cauliflower, broccoli, cabbage, brussels sprout)	In vitro In vivo Human	[143,144,145] [144,146] [[144], [147], [148], [149],^146^]
Vitamin E (Tocopherol)/Fat soluble vitamin		Plant-based oils, nuts, fruits, and vegetables	In vitro In vivo Human	[150,151] [150,151] [[150], [152], [153], [154], [155], [156]]
Curcumin (Diferuloylmethane)/Polyphenol, curcuminoid		Rhizome of *Curcuma longa* (turmeric) and other *Curcuma* spp	In vitro In vivo Human	[157,158,[159], [160], [161],^162^] [[157], [158], [159], [160], [161], [163],^164^,^162^] [157,158,159,[165], [166], [167], [168]]
Rosemarinic acid/ Polyphenol		Culinary herbs (rosemary, mint, sage, basil)	In vitro In vivo	[169,170] [171,172,173]
Oleuropein/ Polyphenol, seco-iridoid		Olive oil	In vitro In vivo Human	[[174], [175], [176], [177], [178]] [[174], [175], [176]] [[174], [175], [176]]
Thymol/ Monoterpenoid phenol		Thyme oil from culinary herb, *Thymus vulgaris*	–	–
Menthol Monoterpene		Oils of Mint (corn mint, peppermint)	–	–
Apiole/ Phenyl propene		Oils of Parsley and celery leaves	–	–
Ursolic acid/ Triterpene		Fruits and herbs (basil, sage, rosemary, thyme, oregano)	–	–
Carvacrol/Mono terpene		Oregano (*Origanum vulgare*)	–	–

Flavonoids, a class of plant and fungus secondary metabolites, are the most common polyphenolic compounds found in the human diet. Flavonoids (including bioflavonoids) are non-ketone polyhydroxy polyphenol compounds with a C-15 skeletal chain, two phenyl groups, and one heterocyclic ring. They include flavanols (catechins), flavonols (kaempferol, quercetin), flavones (apigenin, luteolin), flavanones (hesperidin, naringenin), and anthocyanins [179]. As antioxidants, flavonoids may prevent the progressive impairment of pancreatic β-cell function caused by OS. Dietary intake of flavonoids is associated with biomarkers of insulin resistance and systemic inflammation, such as fasting insulin levels, C-reactive protein, and interleukin (IL)-6 [180]. Intake of specific flavonoids, including quercetin and myricetin, was inversely associated with the risk of T2D [181]. Among flavonoid-rich foods, apple and tea consumption was inversely associated with diabetes risk [182]. Studies have also shown that increased intake of flavonoids and polyphenols in populations above 65 years reduces the relative risk of neurodegenerative diseases [183]. The neuroprotective effect of many flavonoids is associated with their size and membrane permeability, meaning they can cross the BBB, as evidenced by in-vitro diffusion and localization studies [184]. This property justifies the use of flavonoids as dietary supplements and potential drug candidates for neurodegenerative diseases.

### 1.4 Role of specific dietary components

#### 1.4.1 Tea and its components

Green tea contains potent antioxidants such as theanine and catechins, including (−)-epigallocatechin-3-gallate (EGCG) [[185], [186], [187]] that have protective effects against diabetes and associated complications [188] and neurodegenerative diseases [189]. Tea components enhance insulin action and activate the insulin signaling pathway. They also scavenge free radicals and decrease inflammation. Catechins and phenolic acids in tea have better antioxidant effects than the vitamins C, E, and S-carotene in *in*-*vitro* lipoprotein oxidation models [187]. Catechins from tea are absorbed into the blood vessels, which pass the BBB [187].

##### 1.4.1.1 Theanine

Theanine is an ethylamide and an analog of the excitatory neurotransmitter glutamic acid that exhibits L-and D chirality. Tea contains mainly the L-form, which imparts the characteristic tea flavor, and also offers therapeutic advantages for diabetes and hypertension and stress relief [190]. In cell-based studies, l-theanine acts as an insulinotropic agent, partially protecting pancreatic β-cells against OS and restoring the insulin‐secreting ability of the cells [35]. In rat intestinal mucosa, l-theanine upregulates the expression of several transporters responsible for carbohydrate uptake, including acidic, basic and neutral amino acid transporters solute carriers, and intestinal sodium-glucose cotransporter (SGLT)3 and glucose transporter (GLUT)5. At the same time, l-theanine downregulates the expression of proteins involved in fatty-acid transport, including G-protein coupled receptor (GPR)120 and fatty acid-binding protein (FABP)2 [36].

Theanine shows neuroprotective effects may mainly protect against glutamic acid-induced neurotoxicity. An excess of glutamic acid binds to postsynaptic receptors, increasing the membrane's permeability to calcium ions, activating other cellular enzymes, and resulting in neuronal death. Kakuda et al. [189] found that theanine suppressed glutamic acid-induced death of rat cortical neuronal cells and also inhibited the death of hippocampal cornu ammonis (CA)1 and CA3 neurons. The neuroprotective effects of theanine may be associated with its antagonistic effects on glutamate receptors and various glutamate transporters. As such, it may compete with glutamic acid to bind glutamate receptors, thereby suppressing glutamate toxicity [189]. l-theanine also protects against cerebral ischemia in a rat model and prevents brain injury mediated by a glutamate receptor agonist [37]. A randomized controlled trial involving 30 individuals administered 200 mg/d l-theanine for four weeks reported improved mental health by relieving stress and improving cognitive functions [38]. A randomized controlled study including middle-aged and older (50–69 years) Japanese subjects also indicated positive memory and cognitive effects of l-theanine [39].

##### 1.4.1.2 Catechins

Catechins found in green tea inhibit oxidative alterations of low-density lipoprotein and scavenging of active oxygen species such as *O_2_^−^ [189]. Catechins and derivatives include epicatechin (EC), epigallocatechin (EGC), ECG, and EGCG, which act as antioxidants by chelating metal ions.

Green tea catechins improve glucose tolerance. They control hyperglycemia and prevent diabetic complications (reducing risk factors, e.g., OS and obesity) by improving insulin sensitivity, as confirmed *in vivo* and in humans [[40], [41], [42], [45], [46]]. Green tea catechins reduce α-amylase and sucrase activities tested *in vitro* [43]; more specifically, the esterified moiety in EGCG inhibits α-glucosidase activity, studied on rat intestinal extracts [44]. EGCG and ECG reduce glucose uptake and potentially inhibit the sodium-dependent glucose transporter in rat and rabbit intestines [47]. Green tea catechins also minimize glucose absorption by inhibiting gastrointestinal digestive enzymes. Intake of catechin increases glucose uptake in rat skeletal muscle, possibly via GLUT4 translocation [41,42]. Other mechanisms of catechin-induced improvements in glucose tolerance include decreased carbohydrate absorption coupled with increased insulin secretion and sensitivity to maintain glucose homeostasis [41].

The neuroprotective effects of catechins are mediated primarily by preventing lipid peroxidation and radical scavenging. EGCG has better radical scavenging capacities than vitamins C and E [191,192]. The radical scavenging property of EGCG is attributable to its ortho-3′, 4′-dihydroxy moiety or the ortho-trihydroxy group. An increase in the number of hydroxyl groups increases the radical scavenging property. EGCG has a trihydroxy group in the second phenol ring and a galloyl moiety with three hydroxyl groups in the heterocyclic ring, which account for its strong scavenging property [192]. EGCG also regulates tau proteins, specifically suppressing and/or clearing phosphorylated tau isoforms in rat neuronal cells, through a mechanism involving increased expression of adaptor proteins [188]. EGCG reduces amyloid beta (Aβ) and tau toxicity and inhibits apoptosis *in vitro* and in a transgenic AD mouse model [48,49]. Administration of EGCG (100 mg/kg/d) for four weeks in aging rats improves cognitive ability [50]. Recent clinical trials have either been limited or did not report significant therapeutic benefits [51].

#### 1.4.2 Coffee and its components

The antioxidant effects of coffee come from caffeine, cafestol, trigonelline, chlorogenic acid (CGA), melanoidins, quinine, and other phenols such as hydroxycinnamic acid [193,61]. Roasting the coffee seeds partially degrades trigonelline to nicotinic acid and pyridines, which also have antioxidant effects [64].

##### 1.4.2.1 Caffeine

Caffeine content in a cup of brewed coffee (volume ∼450 ml) ranges from 150 to 250 mg of caffeine [194]. The effects of caffeine alone on insulin sensitivity and glycemic control in people with diabetes and healthy controls are negative or controversial. Many studies report that caffeine increases blood glucose levels and decreases insulin sensitivity [53,54]. In contrast, other studies report that caffeine only marginalizes blood glucose levels and does not affect insulin sensitivity [55].

Caffeine lowers the risk of neurodegeneration in AD and PD in humans [56,57] at doses of 3–5 mg/kg/day [56]. It is an adenosine A_2A_ receptor antagonist that augments locomotor activity in animal models; these effects are similar to the effect of A_2A_ receptor inhibitor/blocker theophylline that improves motor function in patients with PD [195]. Caffeine and other adenosine receptor antagonists attenuate dopaminergic neurotoxicity in a mouse model of PD, suggesting such molecules have neuroprotective effects [52]. The role of caffeine/coffee in amyotrophic lateral sclerosis is not very specific. A large longitudinal study including five cohorts ruled out any protective effect [58], while lower risks were reported in a case-control study [59]. However, a recent systemic review, including four metanalysis, reports clinical trials have not been conclusive in PD [60]. Hence, large sample size is possibly needed to achieve discrete conclusions.

##### 1.4.2.2 Trigonelline

Trigonelline is a naturally occurring pyridine alkaloid that is a major constituent of the herb *Mirabilis jalapa* L. and is also isolated from coffee beans and fenugreek. The compound has antidiabetic and neuroprotective effects in addition to its anti-inflammatory, antioxidant, and anti-aging effects [64,196]. It exerts hypoglycemic, hypolipidemic and antioxidant effects, inhibits intestinal glucose uptake, increases glucose tolerance and insulin resistance, and stimulates β-cell regeneration [64]. In human mesangial cells exposed to high glucose, trigonelline reduces cell injury by regulating the Wnt/β-catenin signaling pathway [62], given that this pathway is involved in diabetic neuropathy [65]. In diabetic rats, trigonelline decreases lipid peroxidation, lowers blood glucose and lipid levels, and increases the activity of antioxidant enzymes and insulin sensitivity [197]. In this model, trigonelline normalized levels of SOD, catalase, glutathione, and nitric oxide synthase activities [197]. It improves renal function and renal lesions in diabetic rats [65].

Molecular modeling suggests that trigonelline has a high affinity for the Aβ(1–42) peptide, similar to cotinine, a proposed drug candidate for neurodegenerative diseases, based on docking and *in vivo* studies [63]. In a mouse model, trigonelline protects against lipopolysaccharide-mediated cognitive impairment by reducing OS, inhibiting proinflammatory cytokine levels, and restoring brain-derived neurotrophic factor (BDNF) levels [66]. In this model, pretreatment with trigonelline decreases tumor necrosis factor-α and IL-6 levels and upregulates BDNF levels. Gaur et al. proposed that a standardized hydroalcoholic extract of *Trigonella* fenugreek seed [67] reversed motor symptoms in an animal model of PD by rescuing dopaminergic neurons from toxicity.

##### 1.4.2.3 Cafestol

Cafestol stimulates insulin secretion and increases glucose uptake in human skeletal muscle cells, suggesting this compound could alleviate T2D [68]. Cafestol may protect against diabetes-associated myocardial fibrosis, given that it mitigates against increased collagen synthesis, transforming growth factor-β1 production, and Smad2/3 phosphorylation (seen on exposure to high glucose conditions) in rat cardiac fibroblasts [69]. Cafestol increases the translocation of NRF2 and increases the expression of heme oxygenase (HO)-1, possibly by interacting with Keap1 [69]. Cafestol treatment upregulates the activity of enzymes, including SOD, catalase, and matrix metalloproteinases. It downregulates malondialdehyde levels in a rat model of diabetes with cardiac fibrosis, suggesting it might be used to treat diabetes-related cardiac fibrosis [69]. Antidiabetic effects of cafestol were reported in male KKAy mice (a strain with metabolic abnormalities used for diabetes and obesity research), where cafestol increased insulin secretion from isolated islets by 75–87% [70]. Trinh et al. [71] reported that cafestol has neuroprotective effects in *Drosophila* models of PD, which activates the NRF2/antioxidant response element pathway, increases the expression of HO-1, eliminates excessive ROS production, protects against oxidative DNA damage, and upregulates glutathione [71,72].

##### 1.4.2.4 Chlorogenic acids (5-caffeoylquinic acid), CGA

These are a family of phenolic esters formed in the reaction between *trans*-cinnamic acids and quinic acid (caffeic, ferulic and coumaric acids) that possess hypoglycemic, hypolipidemic, antioxidant, antibacterial, and anti-inflammatory activity [198]. CGA also protects against T2D-induced complications, namely diabetic retinopathy, nephropathy, and peripheral neuropathy [198]. CGA administration (80 mg/kg/d for 12 weeks) improves outcomes in a mouse model of late-onset T2D by modulating adiponectin receptor signaling pathways [75]. The study showed that CGA reduces body fat, fasting plasma glucose, and glycosylated hemoglobin in diabetic mice. In addition, CGA enhances mRNA and protein levels of peroxisome proliferator-activated receptor (PPAR)-α in the liver, increases expression of adiponectin receptors, and increased phosphorylation of AMP-activated protein kinase (AMPK) in liver and muscle [75]. Peng et al. [76] showed that CGA maintains glucose homeostasis by modulating the expression of glucose transporters GLUT-2, SGLT-1, and proglucagon in the intestine of a rat model.

The cognitive and neuroprotective effects of CGA have been reviewed by Heithman and Ingram [199]. Studies show that CGA protects against aluminum-induced neurotoxicity in PC12 cells, where it inhibits the accumulation of ROS and Aβ_1-42_ and decreases apoptosis by regulating the protein kinase B (AKT)/glycogen synthase kinase 3β (GSK3B) signaling pathway [73]. Most notably, CGA protects against Aβ-induced pathology concomitant with AD in mouse models by upregulating the mRNA expression of the glycolytic enzyme phosphoglycerate kinase-1, increasing ATP production [74]. CGA treatment improves motor coordination in a mouse model of PD induced by MPTP (1-methyl-4-phenyl-1,2,3,6-tetrahydopyridine) [77]. In this study, CGA improved the activity of mitochondrial complexes I, IV, and V by enhancing the levels of SOD and mitochondrial glutathione in the brain, inhibited the activation of Bax and caspase-3 (proapoptotic proteins), increased the expression of Bcl-2 (an antiapoptotic protein), and prevented the MPTP-mediated apoptotic cascade. CGA also restored the phosphorylation of Akt, extracellular signal-regulated kinase (ERK)1/2, and GSK3β. Overall, the results suggest that CGA is a therapeutic candidate for mitigating the symptoms of PD caused by mitochondrial dysfunction [77]. A recent study that examined the neuroprotective effects of CGA in hypoxia-ischemia brain injury in neonatal rats showed that the compound activates Sirtuin1 to regulate the NRF2/nuclear factor kappa B (NF-κB) signaling pathway and protects primary neurons from damage induced by oxygen and glucose deprivation [78].

#### 1.4.3 Red wine

Red wine is a rich source of antioxidants, including phenolic acids and polyphenols that protect the redox balance and offer therapeutic advantages [84]. The major phenolic compounds in red wine include catechins, EGC, quercetin, myricetin, caffeic acid, and resveratrol. Although resveratrol is present at lower concentrations than other phenols, it is the major functional compound [200].

##### 1.4.3.1 Resveratrol (3,4′,5-trihydroxystilbene)

Resveratrol is a naturally occurring polyphenol and phytochemical abundantly found in red wine, grapes (particularly red grape skin), berries, peanuts, and chocolate [201]. It has been reported to have a wide range of health-boosting effects, including the prevention of diseases such as diabetes, inflammation, cancer, stroke, neurodegeneration, and aging [85,202]. Resveratrol enhances the expression of many antioxidant enzymes, including glutathione peroxidase, SOD, catalase, and HO-1. It also regulates various signaling pathways, including sirtuin 1, NRF2, and NF-κB, to increase glutathione levels and maintain the cellular redox balance [79,203]. Clinical trials have reported the efficacy of resveratrol in T2D patients, particularly in maintaining glucose homeostasis and insulin sensitivity, as detailed in Refs. [85,86]. In a study of 56 patients with T2D and coronary heart disease, resveratrol increased insulin sensitivity while decreasing insulin levels and insulin resistance compared with placebo controls. The study showed that resveratrol increases the total antioxidant potential, reduces malondialdehyde levels, and upregulates PPAR-γ and sirtuin 1 expression in peripheral blood mononuclear cells of patients with T2D and coronary heart disease [84].

Many studies have reported that resveratrol has neuroprotective effects in AD and PD models and is a potential therapeutic candidate in their management [201,202]. Resveratrol facilitates the non-amyloidogenic breakdown of amyloid precursor protein and the removal of neurotoxic Aβ peptides. It also reduces damage to neuronal cells *via* a variety of additional mechanisms, particularly the activation of NAD (+)-dependent histone deacetylases enzymes (Sirtuins) [79]. Resveratrol induces the differentiation of neurons in cell-based studies and mimics the activity of neurotrophin, suggesting resveratrol may have potential in regenerative medicine applications to help stimulate neurogenesis [80]. Khodaie et al. [81] reported that the combination of ethanol and *trans*-resveratrol at moderate concentrations prevents Aβ neurotoxicity in rat brain cultures, and the synergistic effect was likely due to the action of the combination on the protective antioxidant protein peroxiredoxin-2 (Prx2). Qi et al. [82] propose that resveratrol could have a role in preventing cognitive deficits and neurodegeneration, based on their work in a mouse model of AD induced by intra cerebroventricular injection of Aβ_1-42._ In this mouse model, the activity of the AMPK/peroxisome proliferator-activated receptor-gamma coactivator-1α pathway was increased, and activity of the f NF-κB/IL-1β/NLR family pyrin domain 3 signaling pathway was decreased in the hippocampus and prefrontal cortex. These effects were reversed or recovered by treatment with resveratrol or donepezil (a drug used to improve mental function in people with AD). Resveratrol improved behavioral, biochemical, and histopathological changes in the AD mouse model [82]. The effect of resveratrol on tauopathies (e.g., AD and frontotemporal dementia, in which tau protein aggregates within neurons), including motor function, was investigated in mice overexpressing human tau (JNPL3 P301L mice) [83]. Here, resveratrol reduced the levels of total hyperphosphorylated tau and tended to increase the soluble hyperphosphorylated tau but did not affect motor functions [83].

##### 1.4.3.2 Quercetin

Quercetin is a flavonoid with strong antioxidant properties [204] and is abundant in fruits, vegetables, and other dietary sources. Plant products include tea, coffee, red wine, and foods such as onions, capers, green tea, apples, broccoli, red leaf lettuce, ginkgo, cherries, and elderberry [205,206]. The potential therapeutic advantages include antifungal, anti-bacterial, anti-diabetic, anti-obesity, anti-carcinogenic, and anti-inflammatory effects, cardioprotective and neuroprotective properties and beneficial effects in age-related disorders [207]. Quercetin stimulates AMPK activity in skeletal muscle cells to activate Akt and GLUT4 membrane receptors, facilitating glucose diffusion and metabolism [87]. Quercetin stimulates AMPK activity in hepatocytes and inhibits glucose 6 phosphatase activity [88]. It scavenges ROS and increases the AMP/ATP ratio in pancreatic β-cells, activating the mammalian target of rapamycin and stimulating insulin secretion [89]. Quercetin consumption was negatively related to T2D in a Chinese population (questionnaire-based results), suggesting its protective effect [94]. Oral quercetin (250 mg/d for 8 weeks) improved antioxidant response in T2D patients, while significant changes were not observed in glycemic status and lipid profile [95], while a dose is ≥ 500 mg/day for a period ≥8 weeks reduced the plasma glucose levels, suggesting that the dose and duration of quercetin are crucial determinants of its protective function in T2D [96].

Quercetin is lipophilic, enabling it to cross the BBB to impart neuroprotective effects. It inhibits the formation of Aβ_1-42_ fibrils and oxidative stress in cell-based models [90,91]. Quercetin reduces Aβ1-40 and Aβ1-42 formation and improves cognitive functions in the AD mouse model [92]. Reduced acetylcholine receptors and increased acetylcholinesterase activity in AD cause hyperphosphorylation of Tau protein, reduced secretion of soluble amyloid precursor protein (APP) and increased synthesis of Aβ. Acetylcholinesterase inhibitors restore the neurotransmission between cholinergic neurons. Quercetin inhibits acetylcholinesterase [208], and this property can be helpful to and utilized in considering quercetin as a drug candidate in AD treatment. In rat models, quercetin protects against colchicine-induced cognitive impairment [93]. Quercin also offers therapeutic benefits in PD and HD by reducing oxidative stress and synaptic conductivity and improved cognition [91], evident from cell culture and *in vivo* studies, detailed in Ref. [91]. Clinical trials have also reported that onion (quercetin-rich source) intake for 24 weeks reduced cognitive decline, possibly by reducing depressive symptoms and improving emotional state, compared to placebo food group, in Japanese individuals of age-group 60–79 years [97].

Tea, coffee, and wine are essential dietary beverages with a strong cultural influence. However, scientific advancement and research discovered its therapeutic potential, with precise details on their various components. Many components of the mentioned beverages are involved in reducing OS and improving the pathogenesis of T2D and neurodegenerative diseases by their involvement in multiple cellular pathways or precise targeting of an undesirable chain of events (detailed above) to assist in maintaining health. All the research and its results, based on the various lines of evidence including *in vitro* and *in vivo* studies and clinical trials, will help encourage the discovery and development of specific agents with potential therapeutic values to be further investigated/developed as effective drug candidates, also considering the dose, duration, and mechanism of action and potential targets of these components.

#### 1.4.4 Honey

Honey is a rich source of antioxidants and contains many active constituents, including polyphenols (mainly phenolic acids) and flavonoids (Table 1). These include bio-actives in most honey components (galangin, kaempferol, quercetin, isorhamnetin, and luteolin), while some are limited to specific sources (hesperetin and naringenin). Other antioxidants include benzoic acid, cinnamic acid, chlorogenic acid, caffeic acid, coumaric acid, chrysin, ellagic acid, ferulic acids, gallic acid, myricetin, and syringic acid [5]. Honey shows significant health benefits, including the improved function of organs such as the eye, liver, kidney, heart, brain, pancreas, and testis, in *in vivo* models reviewed in Ref. [5]. In addition to its role in glucose homeostasis (T2D), and neuroprotective and antioxidant effects, it also shows anti-inflammatory, anti-fungal, anti-bacterial, and antihypertensive effects [5,100].

Erujawa et al. [98] investigated the effect of honey as an adjunct to metformin or glibenclamide on glycemic control in a rat model of streptozotocin-induced diabetes. Metformin or glibenclamide combined with honey (four-week treatment) increased insulin secretion, decreased fructosamine levels and improved hyperglycemia, and reduced creatinine, bilirubin, triglycerides levels, and very low-density lipoprotein cholesterol. The effects of metformin or glibenclamide combined with honey were more significant than those of either compound alone or honey alone in diabetic rats [98]. Sirisha et al. [99] studied the effect of honey and insulin in a rat model of diabetic neuropathy. Honey given alone (for six weeks) lowered blood glucose and lipid levels, reduced malondialdehyde levels, and increased total antioxidant levels; this effect was similar to that observed with insulin. The conduction velocity of the sensory nerve improved in rats treated with both honey and insulin but not with either compound alone. A type of honey called ‘mad honey’ (due to potentially toxic diterpenes and grayanotoxins) derived from the nectar of the plant *Rhododendron ponticum* is used as a traditional therapy to manage T2D in eastern Anatolia, Turkey. It has now been proved that mad honey decreases blood glucose and lipid levels, possibly due to the stimulatory effect of grayanotoxins on the parasympathetic nervous system [101]. Low but continuous doses can stimulate insulin secretion in patients with T2D. Studies of mad honey in animal models and clinical trials are reviewed in Bobis et al. [100]. Honey improves lipid profile and reduces CRP and homocysteine levels in patients with hypertriglyceridemia while decreasing the plasma glucose levels in diabetic patients compared to glucose and sucrose intake for 15 days [102]. Diabetic individuals (using metformin) who received 5,15 and 25 g honey daily for four months had reduced HbA1c levels, while triglyceride levels remained unaffected [103]. Total cholesterol decreased in those consuming 5 and 25 g honey [103]. Consumption of honey alone (without accompanied medication for T2D) did not reduce hyperglycemia. However, long term honey consumption (1 year) reduced body weight and hypertension and improved cardiovascular status [104].

Luteolin (a honey polyphenol) protects against microglia-induced neuronal death, prevents hippocampal inflammation, and enhances spatial memory in rats [105]. Luteolin also protects synaptic function. In rat models of neurodegenerative diseases, it restores memory by enhancing basal synaptic transmission and induces long-term potentiation. These effects are achieved by high-frequency stimulation in the dentate gyrus of the hippocampus *via* activation of cAMP response element-binding protein (CREB) [106]. Ferulic acid exerts neuroprotective effects in a mouse model of PD by decreasing the levels of phospho-Akt, phospho-pyruvate dehydrogenase kinase-1, and phospho-Bad and increasing caspase-3 levels [107]. Ferulic acid protects against oxidative stress-associated apoptosis in rats by inhibiting the mRNA expression of intercellular adhesion molecule-1 in rats [28,108]. Kaempferol protects against MPTP-induced PD in mouse models. Oral administration of kaempferol reverses MPTP-mediated behavioral and biochemical changes, including behavioral discrepancies, dopamine exhaustion, reduction of glutathione peroxidase and SOD activities, and elevated malondialdehyde.

Histochemical studies also suggest it inhibits the loss of dopaminergic neurons [28,109]. Treatment of rodents with tulang honey before the induction of kainic acid-induced neurotoxicity prevents neuronal degeneration in the piriform cortex. This result suggests that tulang honey could be therapeutically useful for reducing OS and OS-mediated neurodegeneration [110]. Neuronal differentiation helps in preventing neurodegenerative conditions and aging. Polyphenols such as luteolin, ferulic acid, and kaempferol have the advantages of crossing the BBB and not degrading it in the intestine. They could be used for therapeutic purposes in conjugation with or as mimics of peptide neurotrophic factors (peptide in origin) to promote neuronal growth, differentiation, and survival. Apigenin (which is found in parsley, rosemary, olive oil as well as honey) and resveratrol and apigenin induce neuronal differentiation of murine neuro-2a (N2a) cells through signaling cascades involving protein kinase A/phospholipase C/PKC and ERK pathways; this finding suggests these compounds could have applications in regenerative medicine [80].

Considering the therapeutic benefits of honey *in vitro* and *in vivo* from diverse sources and its wide range of components, further well-planned and focused clinical research would be beneficial in the immediate future. Also, the various component molecules may further be investigated and proposed for therapeutic interventions.

#### 1.4.5 Vitamins

##### 1.4.5.1 Vitamin A/retinol

Retinoids (vitamin A and its analog and metabolites) are crucial antioxidants vital in pancreatic development and the maintenance of and regulation of islet cells. They are involved in hepatic lipid metabolism, adipogenesis, and pancreatic β-cell function. Many vegetables/fruits contain carotenes, which are enzymatically hydrolyzed into retinal and converted to retinol, as are a few xanthines. Retinol is transported from the liver to the peripheral tissues by retinol-binding protein and transthyretin (transport protein), facilitating its cellular uptake together with its membrane receptor STRA6; and have important effects on lipid metabolism and insulin sensitivity [209] in human [115,116] and animal models [116,112]. Consumption of α-/β-carotene and lycopene improves glucose metabolism in T2D patients [210,117], while vitamin A deficiency causes hyperglycemia and loss of pancreatic β-cell mass [211].

Vitamin A controls neuronal differentiation and neural tube formation [138]. Vitamin A and β-carotenes improve OS and cognitive function and reduce toxic Aβ by inhibiting Aβ oligomerization and aggregation in a streptozotocin-induced AD mouse model [138,212]. A biomarker-based clinical study reported an age-related decrease in retinol and its derivatives in the frontal lobe cortex [118]. Retinoic acid regulates the nigrostriatal dopaminergic system [213] and receptor-mediated physiology in the adult brain [214]. Dysregulation of retinoic acid receptor β RARβ, which is involved in transcription, energy metabolism, and neurotransmission via cAMP, G-protein and calcium signaling, is associated with AD, PD and HD [138,113]. The therapeutic effects of vitamin A in MS, including improved astrocyte function and remyelination, were seen in animal models [114] and patients [119].

##### 1.4.5.2 B-complex

Deficiency in many B-complex vitamins — including B1 (thiamine), B3 (nicotinic acid/niacin), B6 (pyridoxine), B7 (biotin), B9 (folate/folic acid) and B12 (cobalamin) — are linked to T2D. Many components of the complex cannot be synthesized by the body and so must come from the diet. Thiamine is essential for antioxidant defense in the mitochondria and cytoplasm and for synthesizing nucleic acid precursors, myelin, neurotransmitters, and lipids [123]. Thiamine deficiency reduces oxidative metabolism and is associated with a failure to produce ATP and defective heme synthesis. Being highly dependent on ATP generation, the central nervous system and heart are very sensitive to thiamine deficiency. People with T2D have lower thymine levels than healthy controls, possibly due to enhanced renal clearance or hyperglycemia-induced tissue impairment. Thiamine supplementation improves blood glucose levels and reduces urinary albumin excretion, possibly reversing early-stage diabetic nephropathy. Thiamine reduces plasma cholesterol and triglycerides in diabetic rats [120] and improves vascular endothelial function in T2D patients [122]. Mice deficient in thiamine have reduced glutathione levels (hence increased lipid peroxidation) and antioxidant enzymes glutathione peroxidase, SOD, catalase, glutathione reductase and glutathione transferase, as well as increased levels of OS markers, including malondialdehyde. The thiamine-deficient mice also have excessive neuronal loss; these results suggest thiamine has a critical role in regulating OS and brain function [121].

**Vitamin B2:** The antioxidant properties of riboflavin promote healing in various models of ischemia-reperfusion oxidative injury, including SH-SY5Y neuroblast cells, rabbit myocardium, and lung and brain injury in rats, reviewed in Ref. [123]. Riboflavin is beneficial in mouse models of T2D via glutathione recovery, reducing blood sugar levels and increasing expression of the GLUT4 transporter [125]. Riboflavin is critical to the proper functioning of mitochondrial pathways, and mitochondrial dysfunction linked to riboflavin deficiency is linked to many neurological conditions, including AD, PD, MS, Alper's syndrome, and Kearns Sayre Syndrome, reviewed in Ref. [126]. It offers neuroprotection by ameliorating mitochondrial function and reducing OS and neuroinflammation *in vitro* and *in vivo*, following antioxidant and anti-inflammatory mechanisms [215,124].

**Vitamin B3:** Available forms of vitamin B3 include nicotinic acid (NA, pyridine-3-carboxylic acid), nicotinamide (NAD, pyridine-3-carboxamide), and nicotinamide adenine dinucleotide phosphate (NADP). Niacin reduces dyslipidemia (a cardiovascular complication in T2D), improves lipid profiles, and achieves target lipid levels in T2D patients [128,129]. NA consumption (1000–3000 mg/day) lowers total cholesterol plasma concentrations and low-density lipoprotein cholesterol. It increases high-density lipoprotein cholesterol levels [130] while reducing total mortality in patients with cardiovascular disease [130] and coronary heart disease [131]. In patients with PD, dietary supplementation with niacin maintains levels of NAD (which is involved in dopamine synthesis) and the expression of nicotinic acid receptor GPR109A. It improves cognitive and motor functions [132]. Niacin also offers therapeutic benefits to patients with AD, PD, HD, MS, and schizophrenia by combating OS, redox imbalance, and mitochondrial dysfunction [123,127].

**Vitamin B6:** Pyridoxal-5′-phosphate (PLP), the active form of vitamin B6 (pyridoxine), aids glycogen metabolism in the liver and muscles [216], regulating the release of glucose. Vitamin B6 supplementation decreased insulin concentrations and sensitivity in an animal model [134]. Pyridoxin and its derivatives inhibit enzymes that hydrolyze and absorb carbohydrates (maltase, sucrase, glucoamylase, α-glucosidases), decreasing blood glucose levels and T2D progression shown *in vitro* and in an animal model [133]. When vitamin B6 is administered with thiamine to diabetic patients, DNA glycation in leukocytes decreases [135]. A six-month clinical trial of vitamin B6 supplementation showed that it decreased retinal edema and increased light sensitivity in diabetic patients with non-proliferative retinopathy [136]. The consumption of PLP, which is involved in synthesizing serotonin and norepinephrine, reduces the risk of T2D, PD, and AD (when consumed with vitamins B9 & B12) [209,137].

**Vitamins B9 and B12:** Deficiencies in folic acid and vitamin B12 are closely linked to T2D pathogenesis; insufficient levels of both these vitamins cause hyper-homocysteinemia in T2D patients [140]. Folic acid and vitamin B12 supplementation improve T2D by reducing OS, reverting DNA damage, improving glycemic control, and reducing serum insulin, insulin resistance, and homocysteinemia [209]. Metformin, a drug of choice for treating T2D, causes folate and vitamin B12 deficiency. Folate and vitamin B12 supplements improve the antioxidant capacity and cognitive functions and reduce homocysteine and malondialdehyde levels (OS marker) [141,142]. Tau aggregation is linked to the activity of cellular kinases and phosphatases. Vitamin B12 acts as a tau protein inhibitor. It impedes the fibrillization of tau protein (by binding to its cysteine residues) *in vitro*. At the same time, its deficiency inactivates protein phosphatase 2 A causing tau hyperphosphorylation and aggregation [139] and the development of neurofibrillary tangles [138]. These effects suggest the critical role of vitamin B12 in AD/tauopathies.

##### 1.4.5.3 Vitamin C (ascorbic acid)

Similar to the vitamin B complex, vitamin C cannot be synthesized in the human body. Key dietary sources of vitamin C include fruits (amla, lemon, orange, star fruit, kiwi, strawberry, guava), vegetables (tomato, potato, cruciferous vegetables such as broccoli and fermented cabbage), and fresh herbs (coriander, parsley) [217]. Vitamin C is a significant source of dietary antioxidants [217]. It prevents endothelial dysfunction [143] by preventing adhesion of leucocytes to the endothelial cells, reducing ROS levels, recovering vasodilation, and decreasing nitrate tolerance; all these effects reduce oxidative stress [143], offering a protective effect on cardiovascular diseases and T2D [144,147]. In T2D patients, vitamin C controls blood pressure, improves glycemic control by increasing insulin synthesis, secretion, and resistance, and negatively regulates HbA1c levels [148,149]. Reduced vitamin C levels are associated with T2D pathology owing to reduced cellular uptake of dehydroascorbic acid (the oxidized transportable form) in red blood cells under high glucose conditions. A high intake of vitamin C overcomes this effect by competing with glucose at the glucose transporters GLUT1 and GLUT3 and increasing its uptake [145]. The beneficial role of vitamin C in neurodegenerative diseases is well-established; it modulates pathways that contribute to cellular homeostasis, neuronal metabolism, and tissue regeneration, thereby maintaining reduced oxidative stress and promoting neuronal health AD, PD, HD, MS, and ALS, reviewed in Ref. [146].

##### 1.4.5.4 Vitamin E (D-alfa-tocopherol acetate)

Vitamin E deficiency affects antioxidant status and glycemic control in T2D. A study of diabetic patients showed a negative correlation between vitamin E deficiency and the glycemic status and lipid profiles [152]. Supplementation in the diet with vitamin E reduces markers of OS (e.g., malondialdehyde and thiobarbituric acid), increases glutathione peroxidase and SOD, changes/improves the total anti‐oxidant capacity and glycemic control, and delays the onset as well as the progression of T2D in patients [153,154]. A combination of vitamin A and vitamin E with zinc improves β-cell function and insulin secretion in T2D patients [155]. Oral supplement of vitamin C and vitamin E (alone and in combination) decreases oxidative DNA damage in diabetic patients [156]. AD patients frequently have low vitamin E concentrations in their cerebrospinal fluid (due to accelerated consumption of vitamin E in the brain/cerebrospinal fluid caused by OS). Vitamin E supplements reduce amyloid deposition and delay AD development, as detailed in Ref. [150]. Karthika et al. proposed that combining natural antioxidants, including vitamin E, quercetin, and basil oil, could be therapeutic in AD [151].

The role of vitamins in cellular health is crucial and diverse. A careful and dedicated screening based on the available investigations may offer potential target-mediated therapeutic candidates for various pathologies.

#### 1.4.6 Curcumin

Curcumin is a pleiotropic polyphenol derived from the medicinal plant *Curcuma longa* that has anti-oxidative and anti-inflammatory properties; it regulates signaling pathways that target inflammatory mediators in diabetes. Curcumin improves β-cell functions and decreases insulin resistance [157]. It inhibits diabetes-related enzymes, including α-glucosidase and aldose reductase, while increasing the activity of antioxidant enzymes. Curcumin improves insulin sensitivity by reducing ERK/c-Jun *N*-terminal kinase-mediated phosphorylation of insulin-resistant cells and activating the phosphatidylinositol 3-kinase-Akt-GSK3B signaling pathway [158]. The anti-diabetic effect of curcumin possibly comes from its ability to suppress OS and the inflammatory process. It targets inflammatory pathways and modulates/normalizes inflammatory mediators (e.g., TNF–α, INF-γ), systemic inflammatory biomarkers (e.g., RANTES), and other inflammatory cytokines [[158], [159], [163]]. Curcumin regulates the expression of keap1 and restores the function of NRF2, promoting insulin sensitivity and redox homeostasis [158,160,161]. A systematic review of 16 clinical studies showed that curcumin reduces the body mass index, fasting blood glucose levels, and HbA1c levels [165]. Curcumin helps reduce diabetic complications in patients by decreasing the serum triglycerides and inflammatory markers, including C-reactive protein, and improving the serum lipid profiles [166,167]. Supplementation of curcuminoids in the diet of T2D patients (1000 mg/day for 12 weeks) promotes the serum total antioxidant and SOD activity and decreases the malondialdehyde levels [168]. Curcumin-based nanoparticle formulations are being extensively investigated for possible therapeutics in many diseases. Nanoparticle formulations of curcumin reduce triglycerides, total cholesterol, very low-density lipoprotein-c, low-density lipoprotein-c, high-density lipoprotein-c, serum CRP, and plasma malonaldehyde, suggesting its potential as a drug candidate in T2D [165]. Curcumin directly binds to and limits aggregation of Aβ; low doses reduce insoluble Aβ plaques by 32% and soluble Aβ by 43% in a mouse model [164]. The protective effect of curcumin against lipid and protein oxidation and activation of antioxidant enzymes, together with its effects on reducing the production of malondialdehyde, protein carbonyls, thiols, and nitrotyrosines — which target multiple molecular targets and signaling pathways — accounts for its neuroprotective effects [162] in several conditions including AD and PD.

#### 1.4.7 Common herbs and oils

Herbs including thyme, rosemary, mint, parsley, basil, oregano, and oils such as olive oil and avocado oil are rich in polyphenols with potent antioxidant properties, with potential therapeutic benefits including protection against T2D and neurodegenerative diseases [2]. Rosmarinic acid is a polyphenol found in common herbs. It ameliorates hyperglycemia and insulin sensitivity in diabetic rats, potentially by modulating the expression of phosphoenolpyruvate carboxykinase, GLUT4 [171], and AKT1 and AKT3 and inhibiting DNA glycation [172]. Rosmarinic acid inhibits the formation of amyloid fibrils *in vitro* [169]. The compact and symmetric structure of rosmarinic acid (Table 1) binds specifically to Aβ, inhibits polymerization into the fibrillar form, and inhibits tau aggregation [170,173], imparting protection in AD and other neurogenerative conditions. Oleuropein is the main phenolic component of olive oil, which has antioxidant and hypolipidemic activities [174]. It has emerged as a novel diabetic nutraceutical. It maintains glucose metabolism by inhibiting several enzymes, including rat and human intestinal maltase and human sucrase, inhibiting glucose transport, increasing incretin release and AMPK activation [175]. Oleuropein inhibits Aβ1-42 peptide oligomerization in AD and tau fibrillization in AD and other tauopathies [176,177]. It also reduces α-synuclein fibrillation and oligomer toxicity in PD [178]. Apigenin is a flavonoid in most herbs, tea, wine, and honey. It manages diabetic symptoms by suppressing the α-glucosidase activity, stimulating insulin secretion, and regulating malondialdehyde, glutathione peroxidase, SOD, and ROS levels. Apigenin acts as an anti-hyperglycemic agent by upregulating the expression of GLUT1 and GLUT4, and regulates the AMPK pathways, improving glucose metabolism. Apigenin has neuroprotective effects in a mouse model of AD (APP/PS1 double transgenic mice), where it improves memory and learning deficits, and reduces fibrillar amyloid deposits by restoring activity of the ERK/CREB/BDNF pathway [111].

Considering the length of the review, we have restricted our inputs about curcumin, common herbs, and oils. These components have been under active investigation recently for potential antioxidative effects and their protective role in various diseases. Thorough knowledge of the yet unexplored sources of many herbs and essential oil and their components may offer better alternatives in augmenting health and preventing diseases.

## 2 Conclusion

Oxidative stress maybe a common cause of various pathologies, including T2D and neurodegenerative disorders, the symptoms possibly advancing with age. Effective drug-based therapy against these critical diseases is crucial. However, complete dependence on specific or multiple drugs is associated with short-term or long-term side effects. The side effects are often associated with reduced absorption, drug resistance and/or non-specific targeting, which are crucial concerns for patients. Using nutraceuticals as dietary supplements and encouraging the intake of foods that directly reduce OS or help the body's intrinsic mechanism to combat OS may alleviate the pathology of or prevent the pathogenesis of neurodegenerative disease and T2D. Many supplements could be used in combination to increase the efficacy of the antioxidant effect. Although a good deal of scientific details is available, many remain inconclusive. A well designed and carefully planned clinical studies/trials incorporating a large sample size is crucial and will be a step toward having discrete results. Thus, a better and comprehensive awareness of the therapeutic potential of dietary antioxidants could lead to better management of chronic diseases; dietary antioxidants might be used as alternatives or in combination with drug-based therapy and could be developed commercially. Moreover, the targets of dietary antioxidants could also be used as pharmacological targets to develop new drug-based therapies.

## Declarations

### Author contribution statement

All authors listed have significantly contributed to the development and the writing of this article.

### Funding statement

This work was supported by the Sidra Medicine, Precision Medicine of Diabetes, Obesity and Cancer program [SDR400175].

### Data availability statement

No data was used for the research described in the article.

### Declaration of interests statement

The authors declare no conflict of interest.

### Additional information

No additional information is available for this paper.

## Abbreviations

AD: Alzheimer's disease
Akt: protein kinase B
ALS: amyotrophic Lateral Sclerosis
AMPK: AMP-activated protein kinase
APP: amyloid precursor protein
Aβ: amyloid beta
BBB: Blood-brain barrier
BDNF: brain-derived neurotrophic factor
c-AMP: c-adenosine monophosphate
CA: cornu ammonis
CAT: Catalase
CGA: chlorogenic acid
CREB: cAMP response element-binding protein
CRP: c-reactive protein
EC: Epicatechin
ECG: epicatechin gallate
EGC: Epigallocatechin
EGCG: Epigallocatechin-3-gallate
ERK: extracellular signal-regulated kinase
FABP: fatty acid-binding protein
GA: glutamic acid
GLUT: glucose transporter
GPR: G-protein coupled receptor
GPx: glutathione peroxidase
GSH: Glutathione
GSK3B: glycogen synthase kinase 3β
HD: huntington's Disease
Keap1: kelch-like ECH-associated protein 1
MPTP: 1-methyl-4-phenyl-1,2,3,6-tetrahydopyridine
MS: Multiple Sclerosis
NA: pyridine-3-carboxylic acid
NAD: Nicotinamide
NADP: nicotinamide adenine dinucleotide phosphate
NADPH oxidase: nicotinamide adenine dinucleotide phosphate hydrogen oxidase
NF-κB: nuclear factor kappa B
NO: nitric oxide
NPE: neuroprotective effects
NRF2: nuclear factor erythroid 2-related factor
OS: oxidative stress
PC12: pheochromocytoma-12
PD: Parkinson's disease
PKC: protein kinase C
PLP: pyridoxal-5’-phospate
PPAR: peroxisome proliferator-activated receptor-
Prxs: Peroxiredoxins
RARβ: retinoic acid receptor β
RNS: reactive nitrogen species
ROS: Reactive oxygen species
SGLT: sodium-glucose cotransporter
SOD: superoxide dismutase
STZ: Streptozotocin
T2D: Type 2 diabetes
TGF-ß1: Transforming growth factor (TGF)-ß1
Trx: Thioredoxin
